# Solventing out crystallization-basic magnesium carbonate percipitation for thorough phosphorus removal from ammonium tungstate solution

**DOI:** 10.3389/fchem.2022.976376

**Published:** 2022-08-17

**Authors:** Liang Yang, Jie Qu, Dandan Gong, Zhongbing Wang, Ruixiang Wang, Linsheng Wan

**Affiliations:** Faculty of Materials Metallurgy and Chemistry, Jiangxi University of Science and Technology, Ganzhou, China

**Keywords:** phosphorus removal, ammonium phosphate, ammonium tungstate, ammonia, basic magnesium carbonat

## Abstract

The ammonium tungstate solution obtained by leaching scheelite with phosphate contains a large amount of phosphorus. For production of qualified ammonium paratungstate products, phosphorus must be deeply removed from the ammonium tungstate solution. In this study, a novel process for ammonium phosphate recovery and deep phosphorus removal from the solution was proposed. First, ammonium phosphate was crystallized and separated from the ammonium tungstate solution by blowing ammonia and cooling. Results showed that the crystallization ratio of phosphorus was above 95% under the conditions of an ammonia concentration of 4.18 mol/L, an initial phosphorus concentration ranging from 15 g/L to 30 g/L, a holding time of 60 min and the temperature of 20°C. Then, the small portion of phosphorus remaining in the ammonium tungstate solution was further deeply removed by basic magnesium carbonate percipitation. The phosphorus removal efficiency was above 99% and tungsten loss was less than 0.22% under the following conditions: the basic magnesium carbonate stoichiometric ratio was 1.5, the initial phosphorus concentration was ranging from 0.5 to 4 g/L, the reaction time was 120 min and temperature was 25°C. After phosphorus removal, the concentration of phosphorus in the ammonium tungstate solution was below 10 ppm, which meant deep phosphorus removal was achieved.

## 1 Introduction

Wolframite and scheelite are the main tungsten resources with industrial application value in nature (Bohlouli et al., 2016; Song et al., 2019; Zhang et al., 2019; Yadav et al., 2020; Liu et al., 2021). Scheelite accounts for more than two-thirds of the total tungsten resource (Dong et al., 2019). Due to the large consumption of wolframite concentrate, scheelite has become the main raw material for tungsten industry (Furberg et al., 2019; Pan et al., 2019; Zhang et al., 2021). Phosphate (ammonium phosphate, sodium phosphate, phosphoric acid, etc.) is an effective leaching reagent for scheelite (Wan et al., 2015; Shen et al., 2019; Zhu et al., 2020). In acid solution, phosphorus and tungsten form soluble phosphotungstic heteropoly acid into leaching solution (Li and Zhao, 2016; Chen et al., 2020). In alkaline solution, phosphate and calcium ions form hydroxyapatite or calcium fluorophosphate with small solubility product, and tungsten enters the leaching solution in the form of tungstate, thus scheelite can be efficiently decomposed (Xiao, 2013; Li et al., 2019). Based on the theory, some new methods of decomposing scheelite have been proposed by metallurgists. Li and Zhao proposed that scheelite was leached by a mixture of sulfuric acid and phosphoric acid, and phosphatotungstic heteropoly acid in the leaching solution and calcium sulfate precipitate were obatined (Li and Zhao, 2016). Crystallized and separated from the leaching solution by cooling crystallization, the phosphotungstic heteropoly acid was dissolved in ammonia water. Then, ammonium tungstate solution containing phosphorus was produced. Researchers put forward that a mixture of ammonium phosphate, ammonia and calcium fluoride be applied to leach scheelite, and ammonium tungstate solution containing high concentration phosphorus was obtained directly (Yang et al., 2018). As an important intermediate product in the tungsten industry, ammonium paratungstate (APT) is usually prepared by the evaporative crystallization of ammonium tungstate solution. According to the relevant state standard (GB/T10116-2007), the content of phosphorus in ammonium paratungstate is lower than 7ppm (Li et al., 2010a). Prior to the evaporative crystallization operation, phosphorus must be deeply removed from the ammonium tungstate solution because phosphorus easily crystallize out in the evaporative crystallization process and affect purity of the ammonium paratungstate products.

Traditional ammonium magnesium salt precipitation method is usually used to remove phosphorus from ammonium tungstate solution (He et al., 2013; Yang et al., 2016). The addition of soluble magnesium chloride to ammonium tungstate solution containing phosphorus produces the magnesium ammonium phosphate precipitation. When the phosphorus content is high, a large amount of magnesium chloride is needed to achieve efficient phosphorus removal, resulting in high concentration of residual magnesium ions in the solution, which causes excessive magnesium content in ammonium paratungstate products (Fu et al., 2018). In addition to magnesium salt, calcium salt is also an effective precipitant of phosphorus, since the solubility product of calcium phosphate precipitation is very small (Ksp = ×210^–29^). However, the calcium salt can also react with tungstate to form calcium tungstate precipitation, resulting in substantial loss of tungsten (Yang et al., 2019). In order to solve this problem, Ji et al. proposed a new method of hydrogen peroxide coordination-calcium salt precipitation to remove phosphorus from sodium tungstate solution. Hydrogen peroxide reacted with tungstate ions to form peroxytungstate ions, thereby avoiding the precipitation of calcium tungstate. At the same time, the calcium salt reacted with phosphate ions to generate calcium phosphate precipitation, accomplishing the efficient removal of phosphorus in the sodium tungstate solution (Ji et al., 2020). Due to high concentration of tungsten in the ammonium tungstate solution, a large amount of hydrogen peroxide is needed to complex tungstate ions. Based on the low solubility product of chromium phosphate, chromium nitrate is used as a precipitant to remove phosphorus from ammonium tungstate solution. Although this method can achieve high phosphorus removal efficiency, the tungsten loss is also large (about 3 *wt*%), and toxic chromium ions is brought into the ammonium tungstate solution (Hu et al., 2018).

Phosphorus in ammonium tungstate solution is not only an impurity, but also a valuable and recoverable element from the perspective of resource recycling. If phosphorus can be crystallized from ammonium tungstate solution in the form of ammonium phosphate, the dual purposes of phosphorus recovery and phosphorus removal can be achieved at the same time. The solubilities of ammonium phosphate and ammonium tungstate present different characteristics in the ammoniacal solution. When ammonia concentration in the solution is high, the effective concentration of ammonium phosphate in the solution is increased due to the strong hydration of ammonia, which can combine a large number of free water molecules (Cao et al., 2004). In addition, with the increase of ammonia concentration, the pH value of the solution increases, and the solubility of ammonium phosphate decreases (Rajesh et al., 2011; Yang et al., 2012; Xu et al., 2019). As a result, the ammonium phosphate solution is easily crystallized out from the ammoniacal solution. Inspired by the principle, we put forward a solventing out crystallization method to recover ammonium phosphate from the ammonium tungstate solution by blowing ammonia. The remaining small portion of the phosphorus in the ammonium tungstate solution is further removed as the form of magnesium ammonium phosphate precipitation with addition of basic magnesium carbonate. The solubility of basic magnesium carbonate is much lower than that of magnesium chloride, so it will not lead to excessive magnesium content in ammonium tungstate solution. Thus, a new process of solventing out crystallization-basic magnesium carbonate percipitation for deep phosphorus removal from ammonium tungstate solution was developed. In the paper, we studied the effects of technological parameters (for instance, ammonia concentration, phosphorus concentration, temperature and dosage of basic magnesium carbonate) on phosphorus removal from the ammonium tungstate solution and determined the optimum processing parameters.

## 2 Experiment

### 2.1 Materials

The ammonium tungstate solution was from a tungsten smelting plant in Ganzhou, Jiangxi province, China. The main composition of the ammonium tungstate solution is shown as Table 1. The reagents used in these experiments including ammonium phosphate, basic magnesium carbonate were analytical pure. Liquid ammonia was provided by Sinopharm Chemical Reagent Co., Ltd. Ammonium phosphate and ammonia were used to adjust the concentrations of phosphorus and ammonia respectively in the ammonium tungstate solution.

**TABLE 1:** Main composition of the ammonium tungstate solution.

Component	WO_3_	P	Mo	NH_3_
Concentration g/L	224.16	5.13	0.38	31.16

### 2.2 Experimental procedure

#### 2.2.1 Separation of ammonium phosphate from ammonium tungstate solution by solventing out crystallization

Experiments of ammonium phosphate separation from the ammonium tungstate solution were carried out in an erlenmeyer flask fixed in a thermostated water bath equipped with a magnetic agitater. 200 ml ammonium tungstate solution containing phosphorus was added to the erlenmeyer flask, then a certain amount of ammonia gas was bubbled into the solution to make the ammonia concentration reached the specified value. After that, the erlenmeyer flask was tightly sealed with a rubber stopper to prevent the ammonia from escaping. At the same time, ammonium phosphate crystals gradually precipitated from the solution. After a prescribed holding time, the slurry in the erlenmeyer flask was filtered. The volume of the filtrate was measured. The concentrations of phosphorus and ammonia in the filtrate were determined. Based on the filtrate volume and phosphorus concentration, the ammonium phosphate crystallization ratio was calculated. The residue was dried at a temperature of 60°C for 4 h. The content of tungsten in the residue was analysed. According to tungsten content in the residue and residue weight, the tungsten loss was calculated. The phase characterization and micromorphology of the residue were analysed with XRD (D/max 2550VB, Rigaku Corporation) and SEM (JSM-6701F). Concentrations of phosphorus, tungsten and molybdenum in the solution were determined by ICP-AES.

#### 2.2.2 Phosphorus removal from the ammonium tungstate solution by basic magnesium carbonate percipitation

After ammonium phosphate was crystallized from ammonium tungstate solution by solventing out crystallization, a small portion of phosphorus remained in the solution. Then phosphorus was deeply removed by adding basic magnesium carbonate. The experiments were also carried out in the erlenmeyer flask fixed in a thermostated water bath equipped with a magnetic agitater. 200 ml ammonium tungstate solution containing phosphorus was added to the erlenmeyer flask, then a certain amount of basic magnesium carbonate was added into the solution. After a prescribed reaction time, the slurry in the erlenmeyer flask was filtered, and the residue was flushed with distilled water. The filter cake was dried at a temperature of 80°C for 4 h. The concentration of phosphorus was determined by ICP-AES. The WO_3_ contents in the residue were measured with a thiocyanate spectrometric method (Zhao et al., 2011). Then phosphorus removal efficiency and tungsten loss were also calculated.

## 3 Results and discussion

### 3.1 Separation of ammonium phosphate from ammonium tungstate solution by solventing out crystallization

#### 3.1.1 Effect of ammonia concentration on crystallization of ammonium phosphate

**FIGURE 1:**
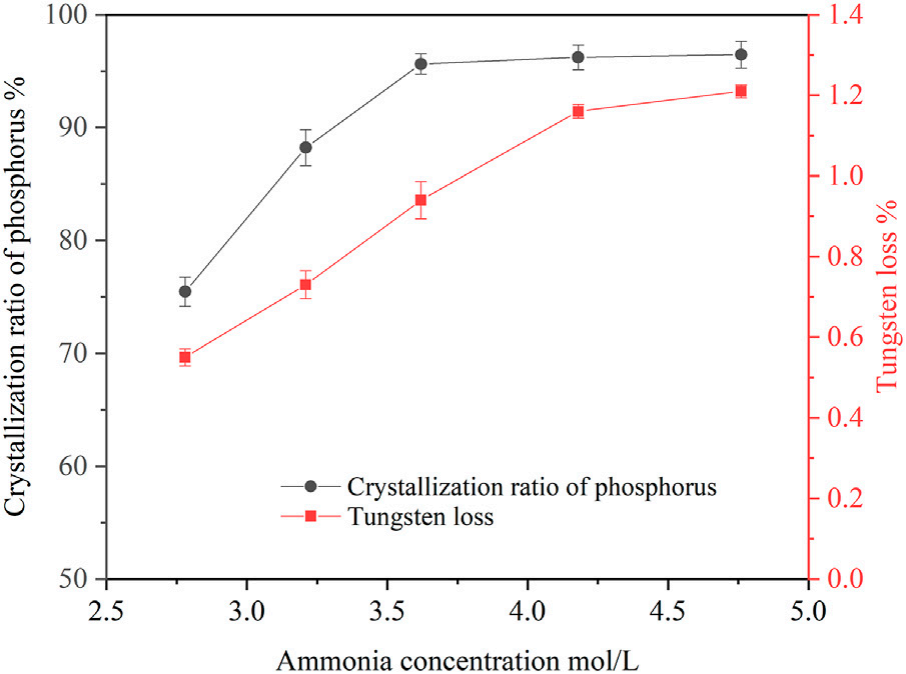
Effect of ammonia concentration on crystallization of ammonium phosphate (P 24.58 g/L, temperature 20°C, holding time 60 min, agitation speed 250 rpm).

Ammonia concentration in the ammonium tungstate solution increased after bubbling ammonia gas into the solution. Ammonia concentration affected the crystallization ratio of ammonium phosphate. The effect of ammonia concentration on the crystallization of ammonium phosphate was investigated under the following conditions: temperature of 20°C, holding time of 60 min, agitation speed of 250 rpm. The concentrations of P and WO_3_ in the ammonium tungstate solution were 24.58 g/L and 224.16 g/L. The ammonia concentration ranged from 2.68 mol/L to 4.76 mol/L, and the results are shown in Figure 1.

As shown in Figure 1, increasing the ammonia concentration improved the crystallization ratio of phosphorus. When the ammonia concentration increased from 2.78 mol/L to 4.18 mol/L, the crystallization ratio of phosphorus rose from 75.46 to 96.23%, and the tungsten loss also increased from 0.55 to 1.16%. With the increase of ammonia concentration, the pH value of the solution increased, which resulted in decrease of ammonium phosphate solubility, thus, phosphorus crystallization ratio increased. Since the precipitated crystals entrained part of the ammonium tungstate solution, the tungsten loss was increased accordingly. When the ammonia concentration was further increased to 4.76 mol/L, the phosphorus crystallization ratio slightly increased to 96.46% and the tungsten loss increased to 1.21%. Considering the phosphorus crystallization ratio and tungsten loss, the optimal ammonia concentration was selected as 4.18 mol/L.

#### 3.1.2 Effect of temperature on crystallization of ammonium phosphate

**FIGURE 2:**
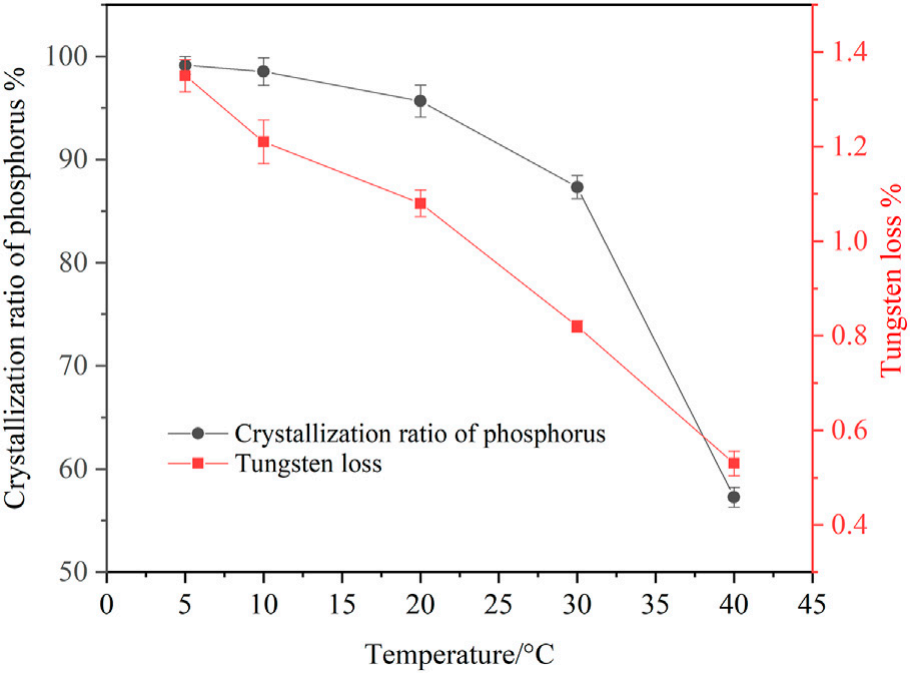
Effect of temperature on crystallization of ammonium phosphate (ammonia concentration 4.02 mol/L, P 24.58 g/L, holding time 60 min, agitation speed 250 rpm).

In general, temperature has a significant effect on the solubility of inorganic compounds in solution. To study the influence of temperature on the crystallization of ammonium phosphate, experiments were conducted within a temperature range of 5–40°C, and the results are shown in Figure 2.

It is shown that temperature had a significant effect on the crystallization ratio of phosphorus from the ammonium tungstate solution. The crystallization ratio of phosphorus decreased from 99.14 to 57.26% when the temperature rose from 5 to 40°C, indicating that decreasing the temperature was conductive to improving the crystallization of ammonium phosphate. However, the tungsten loss increases slightly with the decrease of temperature. When the temperature was 20°C, the crystallization ratio of phosphorus reached 95.67%, and the tungsten loss was only 1.08%. It is inferred that a high phosphorus crystallization ratio can be achieved by dual measures of increasing ammonia concentration and reducing temperature. Considering the feasibility of practical operation, the suitable temperature of ammonium phosphate crystallization was determined to be 20°C.

#### 3.1.3 Effect of initial phosphorus concentration on crystallization of ammonium phosphate

**FIGURE 3:**
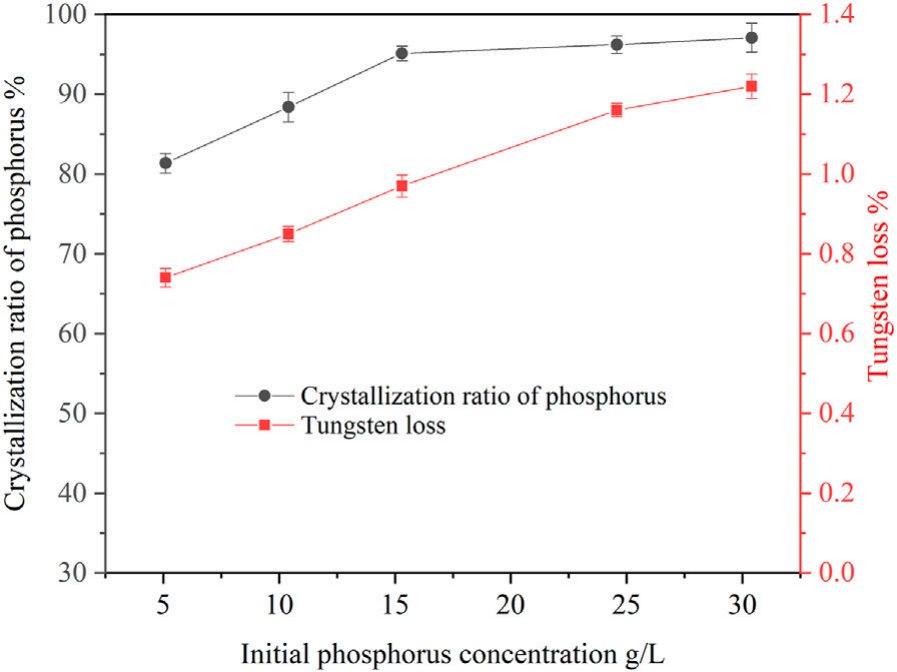
Effect of initial phosphorus concentration on crystallization of ammonium phosphate (ammonia concentration 4.02 mol/L, temperature 20°C, holding time 60 min, agitation speed 250 rpm).

The concentration of phosphorus in ammonium tungstate solution from scheelite treated by different extraction technologies was quite different. Therefore, it is necessary to study the effect of initial phosphorus concentration on the crystallization of ammonium phosphate. In this study, experiments were carried out with initial phosphorus concentration ranging between 5.11 g/L and 30.43 g/L. The results are shown in Figure 3. It can be seen that the crystallization ratio of phosphorus increased with the increase of the initial phosphorus concentration in the solution. When the initial phosphorus concentration was 5.11 g/L, the crystallization ratio of phosphorus was 81.36%. When the initial phosphorus concentration was 30.43 g/L, the crystallization ratio of phosphorus reached 97.05%. Because the solubility of ammonium phosphate was fixed under certain conditions, the crystallization ratio of phosphorus was correspondingly lower when the initial phosphorus concentration was lower. However, when the initial phosphorus concentration was more than 15 g/L, the crystallization ratio of phosphorus was stable at more than 95%, indicating that the new process had a strong adaptability. It can also be seen that the tungsten loss increased proportionally with increasing initial phosphorus concentration. Since the higher the initial phosphorus concentration, the more crystals precipitated from the solution, the higher the tungsten loss due to entrainment.

#### 3.1.4 Effect of holding time on crystallization of ammonium phosphate

**FIGURE 4:**
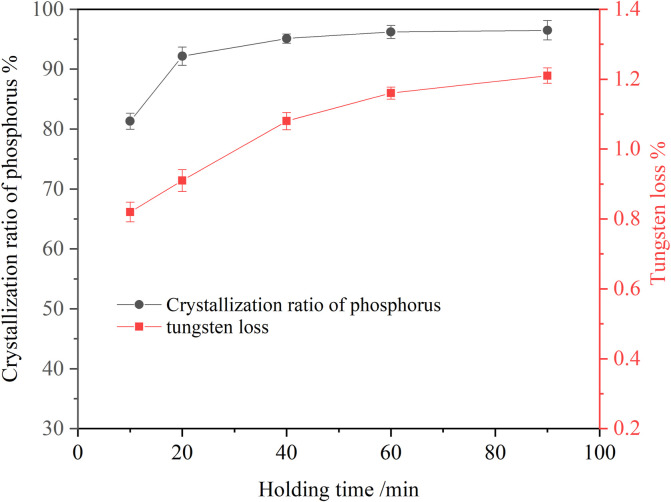
Effect of holding time on crystallization of ammonium phosphate (ammonia concentration 4.18 mol/L, P 24.58 g/L, temperature 20°C, agitation speed 250 rpm).

To explore the effect of holding time on the crystallization of ammonium phosphate, a series of experiments were carried out. The results are shown in Figure 4. It can be seen that in the early stage of the reaction, the crystallization ratio of phosphorus increased rapidly, reaching 81.32% within 10 min. In the later stage of the reaction, the crystallization ratio of phosphorus changed little. When the holding time was 60 min, the phosphorus crystallization ratio reached 96.23%. A further extension of holding time caused no variation in the crystallization ratio of phosphorus. Tungsten loss increased slowly with the extension of holding time. So, the optimum holding time to be 60 min upon taking the phosphorus crystallization ratio and manufacturing efficiency into consideration.

#### 3.1.5 Phase analysis and purification of precipitated crystals

**FIGURE 5:**
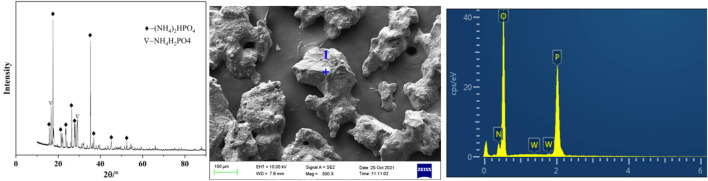
Phase analysis and microanalysis of precipitated crystals.

Under the conditions of phosphorus concentration 24.58 g/L, ammonia concentration 4.18 mol/L, temperature 20°C and holding time 60 min, the obtained crystals separated from ammonium tungstate solution were analyzed by XRD and SEM. As shown in Figure 5 and Table 2, the main component of the crystals was diammonium hydrogen phosphate. In addition, there was a small portion of ammonium dihydrogen phosphate. It is also seen from Figure 5 that the ammonium phosphate crystals were coarse in grain size and contained a certain amount of tungsten. In order to obtain pure ammonium phosphate crystals and recover tungsten, the crystals were washed with ammonia water. The content of tungsten in ammonium phosphate crystal decreases from 1.16 to 0.05%, and the dissolution loss of phosphorus was only 0.54% under the following conditions: ammonia concentration was 5 mol/L, temperature was 20°C, liquid to solid ratio was 3:1, and time was 30 min. Tungsten in the ammonium phosphate crystals was easily dissolved in ammonia solution, which further confirmed that the loss of tungsten during crystallization was caused by entrainment.

**TABLE 2:** Elements content analysis of micro zone 1 of the precipitated crystals (shown in Figure 5).

Element	Mass percentage, %	Atomic percentage, %
N	6.34	9.35
O	46.16	59.59
P	46.39	30.93
W	1.11	0.12

### 3.2 Deep phosphorus removal from the ammonium tungstate solution by basic magnesium carbonate percipitation

Most of the ammonium phosphate was separated from the ammonium tungstate solution through blowing ammonia to increase the ammonia concentration, but a small portion of phosphorus still remained in the solution. In order to prepare high purity ammonium paratungstate in subsequent evaporation crystallization process, the phosphorus in the solution must be deeply removed. In this study, phosphorus was removed as a precipitate of magnesium ammonium phosphate by addition of basic magnesium carbonate. The principle of phosphorus removal is shown as Eq. 1.

$${5\text{PO}}_{4}^{3-}{+5\text{NH}}_{4}^{+}{+\text{ Mg}}_{5}{{(\text{CO}}_{3})}_{4}{\text{·}\left(\text{OH}\right)}_{2\left(\text{S}\right)}\;={5\text{MgNH}}_{4}{\text{PO}}_{4\left(\text{S}\right)}+{4\text{CO}}_{3}^{2-}{+2\text{OH}}^{-}$$ (1)

#### 3.2.1 Effect of basic magnesium carbonate dosage on phosphorus removal

**FIGURE 6:**
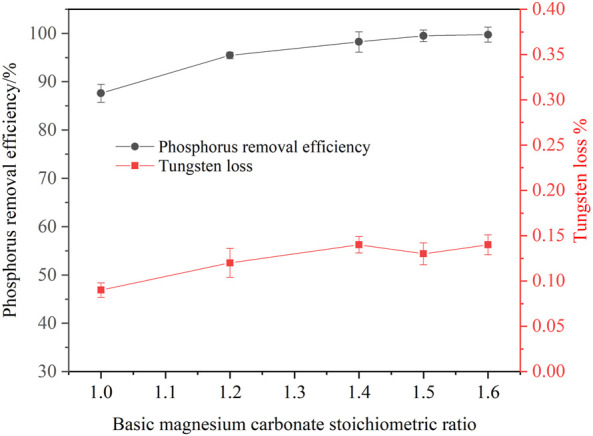
Effect of basic magnesium carbonate dosage on phosphorus removal (P 1.0 g/L, temperature 25°C, agitation speed 250 rpm, reaction time 120 min).

The dosage of basic magnesium carbonate affects the phosphorus removal efficiency in the ammonium tungstate solution. Tests were carried out to investigate the basic magnesium carbonate dosage on the phosphorus removal efficiency under the following conditions: the concentration of phosphorus was 1.0 g/L, the concentration of WO_3_ was 216.37 g/L, the temperature was 25°C, the agitation speed was 250 rpm, the reaction time was 120 min, stoichiometric ratio of basic magnesium carbonate ranged from 1.0 to 1.6. As shown in Figure 6, increasing the basic magnesium carbonate dosage improved the phosphorus removal efficiency. When the basic magnesium carbonate stoichiometric ratio increased from 1.0 to 1.5, the phosphorus removal efficiency increased from 87.61 to 99.5%. A further increase basic magnesium carbonate stoichiometric ratio caused no variation in the phosphorus removal efficiency. In these experiments, the tungsten loss was less than 0.15%. Therefore, the optimal basic magnesium carbonate stoichiometric ratio was 1.5 for ensuring deep phosphorus removal from the ammonium tungstate solution.

#### 3.2.2 Effect of reaction time on phosphorus removal

**FIGURE 7:**
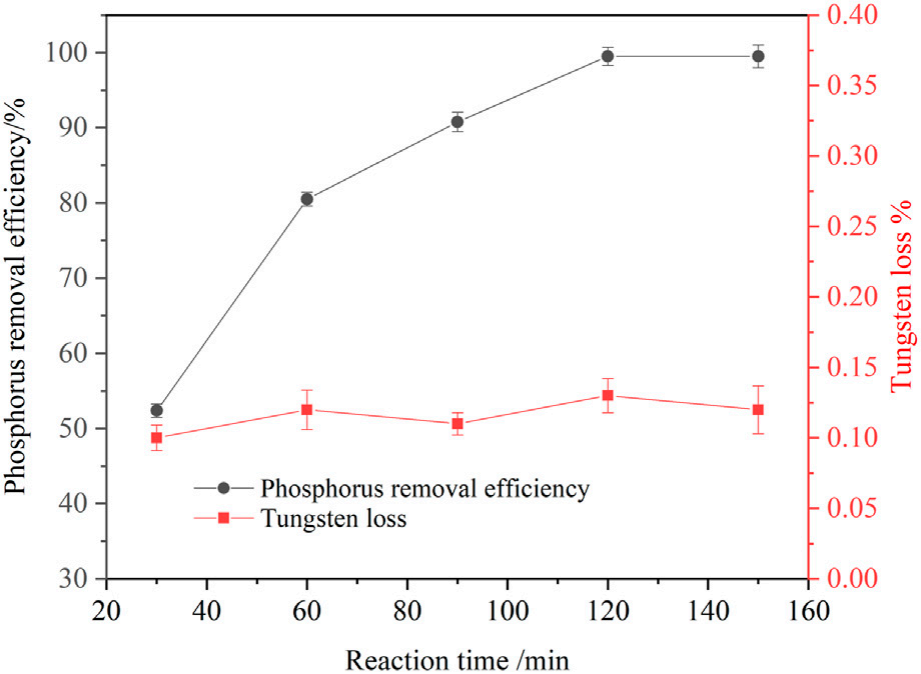
Effect of reaction time on phosphorus removal (P 1.0 g/L, basic magnesium carbonate stoichiometric ratio 1.5, temperature 25°C, agitation speed 250 rpm).

At temperature of 25°C, an agitation speed of 250 rpm, a basic magnesium carbonate stoichiometric ratio of 1.5, effect of the reaction time on the phosphorus removal efficiency was investigated. The results are shown in Figure 7. It is seen that prolonging the reaction time was conducive to improving the phosphorus removal efficiency. With the reaction time increased from 30 to 120 min, the phosphorus removal efficiency increased from 52.37 to 99.50%. Unlike soluble magnesium chloride, the solubility of basic magnesium carbonate in the solution was low. Therefore, it took a long time for basic magnesium carbonate dissolving and releasing free magnesium ions, which formed magnesium ammonium phosphate with phosphorus. When the reaction time was prolonged to 150 min, the phosphorus removal efficiency was almost unchanged. Thus, the suitable reaction time was identified as 120 min.

#### 3.2.3 Effect of initial phosphorus concentration on phosphorus removal

**FIGURE 8:**
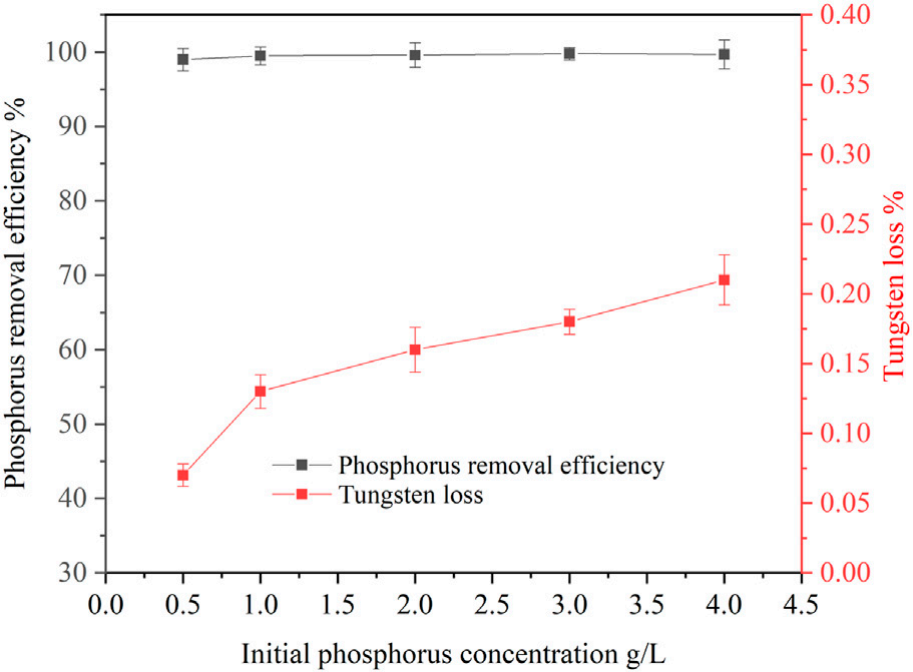
Effect of initial phosphorus concentration on phosphorus removal (basic magnesium carbonate stoichiometric ratio 1.5, temperature 25°C, agitation speed 250 rpm, reaction time 120 min).

The concentration of phosphorus in ammonium tungstate solution is not fixed but in a certain range after ammonium phosphate separation from the solution by solventing out crystallization. Therefore, the effect of initial phosphorus concentration on phosphorus removal was investigated. Figure 8 shows that the phosphorus removal efficiency was high (>99%) and stable in the initial phosphorus concentration range of 0.5 g/L to 4 g/L. This means that basic magnesium carbonate is a good removal reagent for phosphorus, which can achieve deep phosphorus removal, even if there is a high concentration of phosphorus left in the ammonium tungstate solution. However, the tungsten loss increased slightly with increasing initial phosphorus concentration. Because with the increase of initial phosphorus concentration, the dosage of basic magnesium carbonate increased correspondingly, so the tungsten loss caused by entrainment and absorption in the residue was higher.

#### 3.2.4 Effect of temperature on phosphorus removal

**FIGURE 9:**
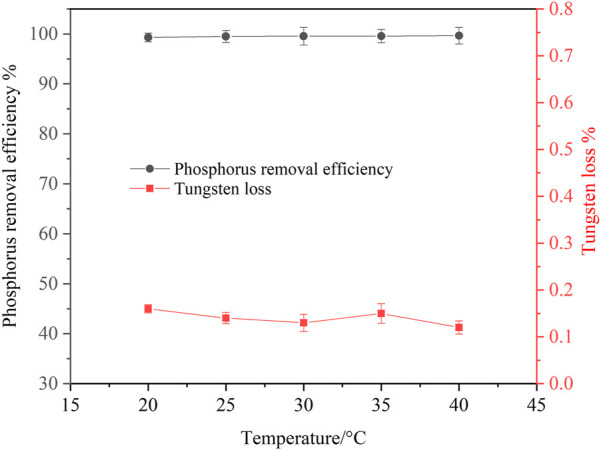
Effect of temperature on phosphorus removal (P 1.0 g/L, basic magnesium carbonate stoichiometric ratio 1.5, agitation speed 250 rpm, reaction time 120 min).

**FIGURE 10:**
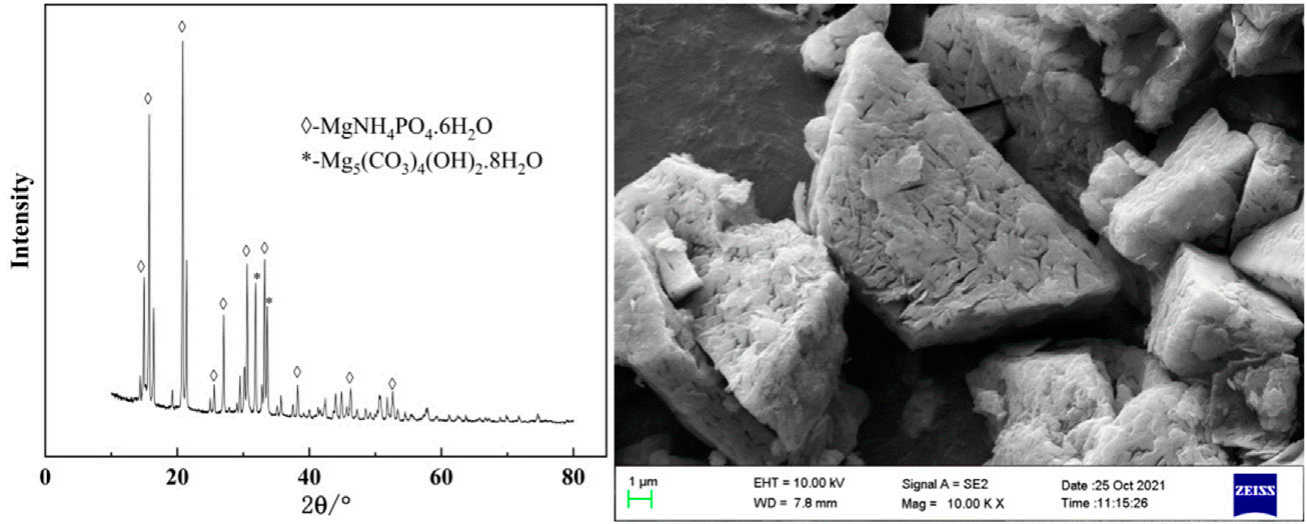
XRD and SEM of the residue.

Experiments for investigating the effect of temperature on phosphorus removal were conducted. Considering that ammonia was easy to volatilize at high temperature, the temperature was ranged from 20 to 40°C. It can be seen from Figure 9 that temperature has little effect on phosphorus removal efficiency and tungsten loss. In the experimental temperature range, the phosphorus removal efficiency remained above 99%. In view of saving energy consumption, the optimum temperature was considered to be 25°C. After phosphorus removal, the main composition of the ammonium tungstate solution is shown in Table 3. The mass ratio of P to WO_3_ in ammonium tungstate solution was only 3.7 × 10^–5^, which can meet the requirement of preparing high purity ammonium paratungstate (APT-0) in the subsequent evaporation crystallization process (Li et al., 2010b). The residue was characterized by XRD and SEM (Figure 10). Results show that the residue was composed of reaction product magnesium ammonium phosphate and unreacted basic magnesium carbonate.

**TABLE 3:** Main composition of the ammonium tungstate solution after phosphorus removal.

Component	WO_3_	P	Mo	NH_3_
Concentration g/L	216.06	0.008	0.32	65.45

## 4 Conclusion

A new process for ammonium phosphate recovery and deep phosphorus removal from ammonium tungstate solution by solventing out crystallization combining with basic magnesium carbonate precipitation has been developed. Ammonium phosphate crystals were efficiently crystallized and separated from the ammonium tungstate solution by blowing ammonia to increase the ammonia concentration and decreasing the temperature. The phosphorus crystallization ratio was more than 95% and tungsten loss was less than 1.2% under the following conditions: ammonia concentration was 4.18 mol/L, temperature was 20°C, holding time was 1 h, and the initial phosphorus concentration was ranging from 15 g/L to 30 g/L. XRD and SEM analysis results indicated that precipitated crystals were diammonium hydrogen phosphate. The diamine hydrogen phosphate was purified by washing the entrained tungsten with 5 mol/L ammonia solution. Upon ammonium phosphate separation, the phosphorus in the ammonium tungstate solution was further deeply removed by addition of basic magnesium carbonate. The phosphorus removal efficiency was more than 99% and tungsten loss was less than 0.22% under the conditions of a basic magnesium carbonate stoichiometric ratio of 1.5, a reaction time of 120 min, a temperature of 25°C, an initial phosphorus concentration ranging from 0.5 g/L to 4.0 g/L. After purification, the phosphorus concentration in the ammonium tungstate solution was below 0.01 g/L, which can meet the requirement of preparing high purity ammonium paratungstate (APT-0) in the subsequent evaporation crystallization process.

## Data Availability

The original contributions presented in the study are included in the article/supplementary material, further inquiries can be directed to the corresponding author.
